# Facing Challenges in Differential Classical Conditioning Research: Benefits of a Hybrid Design for Simultaneous Electrodermal and Electroencephalographic Recording

**DOI:** 10.3389/fnhum.2015.00336

**Published:** 2015-06-09

**Authors:** M. Carmen Pastor, Maimu Alissa Rehbein, Markus Junghöfer, Rosario Poy, Raul López, Javier Moltó

**Affiliations:** ^1^Department of Basic and Clinical Psychology, and Psychobiology, Universitat Jaume I, Castellón, Spain; ^2^Institute for Biomagnetism and Biosignalanalysis, University Hospital Münster, Münster, Germany; ^3^Otto Creutzfeldt Center for Cognitive and Behavioral Neuroscience, University of Münster, Münster, Germany

**Keywords:** emotion, MultiCS conditioning, affective learning, EEG, skin conductance

## Abstract

Several challenges make it difficult to simultaneously investigate central and autonomous nervous system correlates of conditioned stimulus (CS) processing in classical conditioning paradigms. Such challenges include, for example, the discrepant requirements of electroencephalography (EEG) and electrodermal activity (EDA) recordings with regard to multiple repetitions of conditions and sufficient trial duration. Here, we propose a MultiCS conditioning set-up, in which we increased the number of CSs, decreased the number of learning trials, and used trials of short and long durations for meeting requirements of simultaneous EEG–EDA recording in a differential aversive conditioning task. Forty-eight participants underwent MultiCS conditioning, in which four neutral faces (CS+) were paired four times each with aversive electric stimulation (unconditioned stimulus) during acquisition, while four different neutral faces (CS−) remained unpaired. When comparing after relative to before learning measurements, EEG revealed an enhanced centro-posterior positivity to CS+ vs. CS− during 368–600 ms, and subjective ratings indicated CS+ to be less pleasant and more arousing than CS−. Furthermore, changes in CS valence and arousal were strong enough to bias subjective ratings when faces of CS+/CS− identity were displayed with different emotional expression (happy, angry) in a post-experimental behavioral task. In contrast to a persistent neural and evaluative CS+/CS− differentiation that sustained multiple unreinforced CS presentations, electrodermal differentiation was rapidly extinguished. Current results suggest that MultiCS conditioning provides a promising paradigm for investigating pre–post-learning changes under minimal influences of extinction and overlearning of simple stimulus features. Our data also revealed methodological pitfalls, such as the possibility of occurring artifacts when combining different acquisition systems for central and peripheral psychophysiological measures.

## Introduction

Emotional relative to neutral stimuli are preferentially processed and usually attract prioritized attention (Vuilleumier, 2005). The influence of emotion on sensory processing is often investigated by presenting stimuli with intrinsic emotional significance or by assigning emotional value to neutral stimuli in the course of the experiment, as is done during differential classical conditioning. In this paradigm, a neutral stimulus (CS+) is repeatedly paired with an emotional unconditioned stimulus (UCS), while another neutral stimulus (CS−) remains unpaired. Thereby, the CS+ adopts the emotional significance of the UCS and is able to elicit an emotional response (CR), similar to the one evoked by the UCS itself. Traditional differential classical conditioning studies deploy few, often only two CS stimuli (e.g., two line gratings, two faces), and investigate changes in CS+ value by comparing reactions toward CS+ and CS− after relative to before learning. In this regard, research using time-sensitive electro- (EEG) or magnetoencephalography (MEG) showed visual processing of CS+ and CS− to vary after learning during late, mid-latency, and early processing stages [for review, see Miskovic and Keil (2012)]. For example, CS+ relative to CS− faces evoked enhanced positivity over centro-parietal sensors at 464 ms after CS onset in the EEG (Pizzagalli et al., 2003) and enhanced negativity over right occipito-central sensors during 130–180 ms in the MEG (Dolan et al., 2006). Such CS+/CS− differentiation appears similar to the *late positive potential* (LPP) and the *early posterior negativity* (EPN), two components usually found in processing of emotional relative to neutral pictorial stimuli [e.g., Schupp et al. (2000, 2006, 2004a,b, 2003), Junghöfer et al. (2001), and Keil et al. (2002)]. Stolarova et al. (2006), Keil et al. (2007), as well as Hintze et al. (2014) revealed enhanced neuronal activity for simple gratings or Garbor gratings (CS) associated with aversive compared to neutral UCSs already before 100 ms. However, such rapid differentiation of CS+ and CS− stimuli within the first 100 ms after stimulus onset also occurs for more complex facial stimuli [e.g., Steinberg et al. (2013a, 2012)]. In addition, reports of neuronal CS+ enhancement are often paralleled by increased autonomic reactivity toward the CS+, as revealed by studies measuring skin conductance [e.g., Flor et al. (1996) and Hermann et al. (2000, 2002)].

Psychophysiological investigations using differential classical conditioning face several challenges. First, in EEG/MEG, meaningful brain responses evoked by conditioned stimuli are typically intertwined with ongoing background brain activity, from which it is separated due to its stimulus-locked occurrence on each of many trials. Thus, uncovering differences in CS+/CS− processing requires sufficient signal-to-noise ratio, for which typically many repetitions of stimuli in each condition are necessary. However, more often CSs are repeated after learning, the greater is the extinction of the conditioned response (CR) and the lower the expectancy that a UCS will follow the CS+ [e.g., Rescorla and Wagner (1972) and Miller et al. (1995)]. A solution could be provided by not only comparing emotional responding to CS+/CS− after relative to before conditioning but also to assess emotional responses while learning is still ongoing (i.e., during the acquisition phase). For example, recent studies using steady-state visual evoked potentials (ssVEPs) revealed amplified cortical processing of CS+ relative to CS− stimuli to appear already during acquisition of CS+/UCS contingencies [e.g., Keil et al. (2013), Miskovic and Keil (2013), and Wieser et al. (2014)]. Unfortunately, assessment of additional dependent measures, especially of electrodermal activity, during the acquisition phase is technically difficult to carry out simultaneously with EEG/MEG, because responses to the CS+ can be influenced by the occurrence of the UCS (e.g., electric shock, aversive noise) if the UCS is not sufficiently delayed in time. Disturbances by UCS occurrence could be avoided using partial reinforcement designs [e.g., Pizzagalli et al. (2003) and Dolan et al. (2006)], in which the CS+ is reinforced on a given percentage (e.g., 50% contingency) of trials only. Accordingly, responses to the CS+ might not be affected by UCS occurrence in the above studies, because only unreinforced CS+ presentations during acquisition trials enter the analysis. Still, influence of extinction will be minimal, as a CS+ without UCS association does not predict that the following CS+ presentation will also be unpaired – as it is the case in the extinction phase without further CS+/UCS pairings.

The above methodological approach, however, might not be an optimal choice for some research questions. For example, trauma-related research might investigate recall of previously acquired emotional memory and not fear reactions during memory acquisition, which is why it may require a pre–post-conditioning (learning, training) instead of a partial reinforcement design. Thus, pre–post designs are needed, during which many CS+/CS− repetitions ensure a good signal-to-noise ratio, but extinction of the previously acquired CS+/UCS and CS−/noUCS associations is low. Second, if only few CSs are associated with UCS or noUCS during learning, neuronal CS+/CS− differentiation could easily rely on low-level information from simple features only (e.g., line orientation, head shape), instead of relying on more complex high-level information (e.g., facial identity). Such *overlearning* [cf., Steinberg et al. (2013a), 237 p.] was suggested to result in reduced electrophysiological differentiation, with observable variation in CS+/CS− processing during early, but not mid-latency or late processing stages [cf., Steinberg et al. (2013a)]. Third, investigations of specific research questions in affective neuroscience can strongly profit from the simultaneous measurement of different dependent variables, as emotions are complex phenomena, often inducing characteristic changes on various response measures [e.g., Gazendam et al. (2013)]. The parallel investigation of several response measures may thus foster specific interpretations while a single measure may erroneously reduce true complexity. Changes in skin conductance activity (SC) index autonomic fear learning in classical conditioning paradigms. In contrast to amplified CS+ potentials (EEG) or magnetic fields (MEG), enhanced SC responses toward the CS+ develop slowly (i.e., several seconds after CS+ onset), which is why this measure requires quite long trial durations and extended inter-trial intervals (ITIs). Simultaneous SC and EEG/MEG recordings thus often pose a methodological challenge, as a great number of repetitions per condition – toward a good signal-to-noise ratio – needs to be reconciled with a sufficient trial duration (autonomic recovery), while not overstraining alertness of human participants.

Certain adjustments of traditional classical conditioning designs could serve for overcoming these difficulties. Excellent signal-to-noise ratio with minimal extinction could be achieved by increasing the number of CSs in the paired (CS+) and unpaired (CS−) conditions. The greater the number of different CS+ and CS− stimuli is, the less often the individual stimulus has to be presented for achieving the necessary repetition of conditions. Such a procedure was named MultiCS conditioning [see Steinberg et al. (2013b), for more information), and successfully revealed neuronal CS+/CS− differentiation in several MEG and EEG investigations of auditory, olfactory, and electric-shock conditioning (Bröckelmann et al., 2013, 2011; Steinberg et al., 2012; Rehbein et al., 2014b). If, in addition to increasing the number of CSs, the number of learning trials is reduced, neuronal capacities of stimulus differentiation are challenged and overlearning of stimulus features impeded. Although results suggest that participants are able to differentiate many different CSs – at least with regard to neuronal measures of implicit or unconscious stimulus processing with limited contingency awareness (Bröckelmann et al., 2011, 2013; Steinberg et al., 2012; Rehbein et al., 2014b) – experimenters should not use too many stimuli, if contingency aware and thus autonomic fear learning is to be studied (Hamm and Weike, 2005). For simultaneous central EEG/MEG and peripheral SC measurement many trials with short and long durations have to be integrated, so that sufficient signal-to-noise ratio and autonomic recovery are guaranteed.

In the current study, we developed and evaluated an experimental set-up, which incorporates the three above proposed modifications in differential classical conditioning designs (i.e., increased number of CSs, reduced number of learning trials, and inter-mixed trial duration with short and longer ITIs). In this experimental set-up, four CS+ faces with neutral expression were paired four times with an aversive electric stimulation, while four CS− faces with neutral expression remained unpaired. Faces were presented 20 times each before and after learning with varying trial duration. We investigated to what extent CS+/CS− differentiation as observed in partial reinforcement [e.g., Pizzagalli et al. (2003) and Dolan et al. (2006)], or peripheral-physiological designs [e.g., Hermann et al. (2002)], would emerge in this hybrid new approach. More specifically, after relative to before learning, we expected to observe increased autonomic (electrodermal) reactivity during the CS+ compared to the CS−, besides enhanced CS+ vs. CS− cortical processing, as indexed by the LPP, EPN, and other early event-related potential (ERP) components (<100 ms). In addition, we hypothesized that participants would be aware of CS+/UCS contingencies at the post-experimental query, and CS+ faces would be rated as less pleasant and more arousing than CS− faces after conditioning. Furthermore, we evaluated whether the change in subjective valence and emotional arousal of CS+ and CS− faces would be strong enough to bias evaluative ratings of angry and happy facial expressions of the respective CS+/CS− face identities into the direction of conditioning. Indeed, this evaluative task was included as additional validation that any changes in CS hedonic valence relied on an association of CS identity and UCS occurrence, and not on CS low-level features and UCS occurrence. Thus, the current study could be considered a precursor to a follow-up investigation concerning the interaction of affective identity and facial expression (Rehbein et al., 2014a). To this aim, participants also took part in a second EEG experimental task (results not reported here), in which the same CS+ and CS− (i.e., *a priori* neutral faces) were presented again – but intermixed with angry and happy emotional expressions – for the same identities embedded in the CSs.

## Materials and Methods

### Participants

Forty-eight students of Psychology undergraduate studies from Universitat Jaume I (Castellón de la Plana, Spain), aged between 20 and 42 years (*M* = 24.4, SD = 5.1), participated voluntarily and obtained credit for participation. They received detailed information about the experiment in oral and written form before participation and gave written informed consent to the procedure approved by the university Institutional Review Board. None were undergoing psychiatric or pharmacological treatment, and none presented visual or auditory deficits, as confirmed with a semi-structured interview at the beginning of the session. From overall analysis, two participants were excluded due to computer failure and one because of high tolerance to the electric shocks used as unconditioned stimuli (i.e., shock intensity was set to more than three times the interquartile range of intensities of all subjects). Furthermore, nine participants had to be excluded due to low reliability in post-experimental contingency report. As differential SC responses have been shown to be highly dependent on contingency awareness [e.g., Montañés et al. (2004) and Pastor et al. (2013)], we only included participants with a minimum sensitivity of 83.33% (i.e., who were able to identify 75% of all CSs correctly as CS+ or CS−). In EEG only, one additional participant was excluded due to problems with recording. Thus, the final sample consisted of 36 participants (9 men) for analysis of evaluative ratings and contingency reports, and of 35 participants (9 men) for EEG analysis. For SC changes, 2 additional participants were excluded due to high reactivity (outliers) or problems during recording, leaving 34 participants (8 men) in these analyses.

### Stimuli and materials

#### Conditioned Stimuli

Eight different neutral faces (four CS+, four CS−) from the NimStim set of facial expressions (Tottenham et al., 2009) were selected as conditioned stimuli. The specific four faces that served as CS+ or CS− were counterbalanced across subjects, who were randomly assigned to the two versions of the task. The pictures were displayed on a 22″ LCD TFT monitor using Presentation software (Neurobehavioral Systems), with a maximum size of 13.5 cm × 16.5 cm, presented approximately 1.25 m from the participant’s eyes with a visual angle of 6.2° horizontally and 7.6° vertically. For subjective affective ratings task only, 16 additional faces were used, showing the 8 CSs with angry or happy facial expression to have a broader range that facilitated ratings. Thus, affective ratings were performed across a total of 24 items, which corresponded to 8 different individuals (4 CS+, 4 CS−) displaying 3 different emotional expressions (angry, neutral, happy) each.

#### Unconditioned Stimulus

The UCS consisted of a 500-ms train of 0.5 ms electric pulses presented at a rate of 64 Hz. It was generated with a Digitimer DS7A High Voltage Stimulator, which provides constant current as well as high-voltage pulses of brief duration for percutaneous stimulation during experimental investigations and which complies with the guidelines for clinical use issued by the European Community. The UCS was administered during the last 500 ms of each CS+ presentation in the acquisition phase via an electrode attached on the inside of the upper right arm (*n* = 21; five men) or upper left arm (*n* = 15; four men). A workup procedure was used prior to conditioning to set UCS intensity individually to a *highly annoying, but not painful* level [e.g., López et al. (2009)]. The mean intensity used in this experiment was 4.32 mA (Min = 2.8 mA; Max = 7 mA).

### Experimental procedure and design

**Figure 1:**
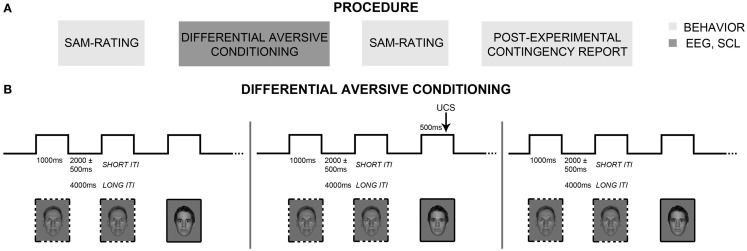
**Experimental procedure and differential classical conditioning**. **(A)** The experimental procedure consisted of three different parts: The first and third parts included evaluative (SAM-ratings) and behavioral (post-experimental contingency report) measures of classical conditioning (boxes in light gray). In the second part, differential aversive conditioning was carried out, while electroencephalography (EEG) and skin conductance changes were recorded (box in dark gray). **(B)** Differential aversive conditioning consisted of three continuous phases (habituation, acquisition, extinction). During habituation and extinction, all conditioned stimuli (CSs) were shown without presentation of the aversive electric stimulation (UCS). During acquisition, four of the eight neutral faces (CS+; solid frame) were paired four times each with the UCS, while the other four neutral faces (CS−; dashed frame) were also shown four times, but never paired with the UCS. During all three phases, trials were shown with either short or long inter-trial intervals (ITIs) to allow for multiple repetitions in EEG but also for sufficient autonomic recovery. Note that in accordance with NimStim publication guidelines, only one model (#28) from the NimStim database is displayed. The other image shows one of the authors, but was not used as stimulus in the present study.

The experiment consisted of three different parts (Figure 1A). Evaluative and behavioral indices of associative learning were measured before and immediately after a differential aversive conditioning task, which comprised habituation, acquisition, and extinction phases. Central (EEG) and autonomous (SC) nervous system correlates were measured during aversive conditioning.

#### Subjective Ratings of Valence and Arousal

First, participants rated the hedonic valence and emotional arousal of all 24 faces (8 individuals with angry, neutral, or happy expression) using a paper-and-pencil version of the self-assessment manikin (SAM; Bradley and Lang, 1994). Evaluative ratings were expressed on a scale from 1 to 9 for each dimension, with higher numbers indicating higher pleasantness or arousal, respectively. Subsequently, participants proceeded with the differential aversive conditioning task. Immediately after the conditioning task, participants again rated the hedonic valence and emotional arousal of all 24 faces.

#### Differential Aversive Conditioning

Sensors for physiological recording (EEG, SC) were attached to participants reclining in a comfortable armchair located in a dimly lit, sound-attenuating room. After setting UCS intensity using the workup procedure (see above), participants were instructed to look at the faces the entire time they were on the screen, and to ignore occasional electric shocks and noises presented over headphones. Differential aversive conditioning consisted of three phases (Figure 1B): habituation (160 trials), acquisition (32 trials), and extinction (160 trials). During each phase, series of four CS+ and four CS− faces (eight individuals with neutral expression only) were presented in a pseudorandom order with maximally three consecutive presentations of each CS type. Duration of each CS presentation was 1000 ms. Habituation and extinction consisted each of 20 CS series including 4 CS+ and 4 CS− with different randomization. For habituation and extinction, a mean ITI duration of 2 s (range: 1.5–2.5 s) was used for 100 trials, and was increased to 4 s for the other 60 trials in order to record 20 measurements of electrodermal changes in each phase (for more information, see Skin Conductance Measures). During acquisition, four CS series consisting of four CS+ and four CS− trials were used and the UCS appeared during the last 500 ms of each CS+. Here, mean ITI duration was 2 s (range: 1.5–2.5 s) for 20 trials, and was increased to 4 s for the remaining 12 trials in order to record 4 SC responses (2 CS+, 2 CS−). These SC changes during acquisition are not reported, because of the elevated number of artifacts due to the early shocks appearance during CS+ presentation and the clearly insufficient number of recorded acquisition trials. After extinction and the second valence and arousal ratings, participants performed a post-experimental contingency query.

#### Post-Experimental Contingency Report

Participants completed a forced choice assignment including all eight CS faces used during aversive conditioning (eight individuals with neutral expression). More specifically, they were asked to indicate for each CS whether it had or had not been paired with the shock at any time during the experiment. In addition, participants rated their level of confidence on a scale ranging from 0 (*not confident at all*) to 100 (*absolutely confident*). This post-experimental contingency query served for assessing whether participants had learnt the CS+/UCS association.

Afterwards, participants took part in a second EEG measurement, then were moved to a new experimental room, and completed several personality questionnaires (all these results not reported here). Participants were debriefed and thanked for their collaboration. The entire procedure lasted approximately 2 h.

### Skin conductance measures

#### Data Acquisition and Reduction

Stimuli control and physiological data acquisition were accomplished using a Compaq V70-compatible computer (VPM software: Cook, 2001). SC activity was recorded through two Ag/AgCl standard electrodes (K-Y lubricating jelly) placed on the thenar and hypothenar eminences of the left (*n* = 21; five men) or the right (*n* = 13; three men) hand palm. For all subjects, UCS presentation electrode and SC recording electrodes were positioned contralaterally. A constant voltage (0.5 V) was generated between the electrodes using a Coulbourn V71-23 Isolated Skin Conductance Coupler. Activity was acquired at 20 Hz, and half-second bins of mean SC were calculated offline. SC changes were recorded 40 times throughout the experiment (10 CS+, 10 CS− during habituation; 10 CS+, 10 CS− during extinction)^1^. For each of these 40 trials, the sequence started with a CS− followed by a 4 s ITI (to guarantee a stable baseline), then continued with a CS− and then with a CS+ (the target stimuli to examine whether there was electrodermal discrimination), both of them with 4 s ITI. Another 20 trials with 4 s ITI were included as “fillers” within the experimental sequence, only for avoiding possible predictions when detecting a longer interval but not for analyses purpose. The sequence for these additional trials was a CS− followed by a CS+ and then another CS+. For assessing reactions to CS+/CS-, SC change scores (ΔμS) were calculated for each half-second bin by taking the respective mean activation and subtracting a pre-trial 1-s baseline (i.e., averaged activity during 1-s directly preceding target CS onset). According to the extended prior literature [cf., Bradley et al. (2001)], the maximum change score between 1 and 4-s after CSs onset was taken as the selected parameter for statistical analysis. Logarithms of raw scores (log [change + 1]) were computed for statistical analyses.

#### Statistical Analyses

Overall mean SC changes were analyzed with a repeated measure ANOVA including the factors *Phase* (Habituation, Extinction) and *CS Type* (CS+, CS−). Because the expected CS+/CS− differentiation after learning was not found when looking at the overall mean change score, we examined SC changes during extinction more closely. Here, we aimed to explore “when” plausible conditioning effects were extinguished after contingency removal. More specifically, *t*-tests compared the SC activity for each measurement (CS+ vs. CS−) conducted during extinction separately, starting with the first and progressing to the consecutive measurements, taking into account that those SC measurements were not necessarily consecutive along the experimental sequence (see text footnote 1). CS+/CS− differentiation was significant only during the first two measurements of extinction, and mean CS+/CS− change scores during these first two extinction measurements were compared against CS+/CS− differentiation during the last two measurements of habituation to keep the same number of trials across phase. Hence, an additional repeated measures ANOVA including the factors *Phase* (Habituation, Extinction) and *CS Type* (CS+, CS−) was carried out to assess whether there was (or not) CS+/CS− differentiation across time before affective learning. Statistical analyses of SC changes were carried out with StatView software (SAS).

### Electroencephalography

#### Data Acquisition and Preprocessing

Event-related potentials in a frequency range between 0.1 and 100 Hz were recorded continuously with a sampling rate of 250 Hz using a 128-channel Electrical EEG system (Geodesics). During recording, the vertex sensor (Cz) was used as reference and all impedances were kept below 50 kΩ. Offline, visual evoked potentials (VEPs) were filtered using a high-pass frequency of 0.1 Hz and a low-pass frequency of 40 Hz. Epochs with a duration of 800 ms (200 ms before to 600 ms after CS onset) were extracted, aligned to CS onset, and baseline-adjusted using a 150 ms baseline interval, ranging from −150 to 0 ms (i.e., CS onset). Single trials and artifacts were edited using the method for high-density EEG/MEG data proposed by Junghöfer et al. (2000). Subsequently, epochs were averaged in correspondence to *Phase* (Habituation, Extinction) and *CS Type* (CS+, CS−). The number of remaining trials [Habituation CS+: *M* = 62.7, SD = 8.0; Habituation CS−: *M* = 62.8, SD = 8.0; Extinction CS+: *M* = 53.5, SD = 11.3; Extinction CS−: *M* = 53.9, SD = 10.7] differed between phases, *F*(1, 34) = 48.41, *p* < 0.001, but not between CS types, *F*(1, 34) = 0.12, *p* = 0.733, and there was no interaction of both factors, *F*(1, 34) = 0.06, *p* = 0.801. For each CS Type, pre-learning (i.e., habituation) activation was subtracted from post-learning (i.e., extinction) activation, so that differences in CS+/CS− processing after learning could be assessed independently of potential differences in baseline (i.e., habituation) activation. The following analysis was based on these difference activations (i.e., the terms ΔCS+ and ΔCS− refer to the difference of extinction minus habituation activation calculated for each CS type).

#### Analysis

We investigated whether the typical centro-parietal positivity for ΔCS+ relative to ΔCS− would emerge during the LPP time interval, which would indicate successful affective learning. The LPP time interval was specified based on existing literature and the global power of the grand average to range from 300 to 600 ms after CS onset (Figure 3A). Originally, we had also been interested in differential CS+/CS− activation during the EPN and earlier time intervals (<100 ms). However, we refrained from analyzing these earlier components, as shifts of the common ground (ground for all applied stimulation and detection devices) revealed condition dependent amplitude variations of uncertain cause^2^, which affected effects in the EPN and preceding time intervals. We thus restricted EEG analysis to LPP because this sustained component reflected stable learning-related changes in CS+/CS− processing beyond doubt. LPP analysis consisted of two steps which incorporated non-parametric correction for multiple comparisons proposed by Maris and Oostenveld (2007): (1) A *t*-test comparing *CS Type* (ΔCS+, ΔCS−) was calculated across all sensors and time-points, due to which every sensor was assigned its respective *t*-value for the difference in ΔCS+ and ΔCS− activation at each time-point of measurement. Subsequently, only those sensors and time-points which surpassed the critical positive *t*-value equivalent to *p* < 0.05 within the LPP time interval (sensor-level criterion) were considered. This directed testing approach was adopted because we expected a positive difference in ΔCS+ vs. ΔCS− activation with a very specific topography and latency. (2) Sums of *t*-values (i.e., cluster masses) were calculated for all neighboring sensors exceeding the sensor-level criterion for at least five consecutive time-points. Cluster masses were compared against a distribution-independent positive *t*-value (*p* < 0.05) established using Monte Carlo simulations of 1,000 random permutations of the whole data set (cluster-level criterion). Significant cluster masses were subjected to parametric *post hoc* analyses, to corroborate that a potentially significant effect stemmed from a difference in CS+/CS− processing after (i.e., extinction), but not before conditioning (i.e., habituation).

Preprocessing and analysis were conducted using the Matlab-based EMEGS software (Peyk et al., 2011; www.emegs.org). Continuative *post hoc* analyses were carried out with SPSS Statistics (IBM).

### Behavioral and evaluative measures

#### Post-Experimental Contingency Report

Contingency awareness of the final sample (*N* = 36) was described by percentage and confidence of correctly matched CS+/UCS and CS−/noUCS pairs. Success of matching and level of confidence were compared between CS+/UCS and CS−/noUCS pairings using *t*-tests. Non-parametric sensitivity index *A*′ (Grier, 1971) was estimated and compared against a chance level of 0.5 using a one-sample *t*-test.

#### Subjective Ratings of Hedonic Valence and Emotional Arousal

To assess how conditioning influenced evaluative ratings toward CSs (i.e., CS+/CS− differentiation), separate repeated measures ANOVAs including the factors *Session* (Pre-conditioning, Post-conditioning) and *CS Type* (CS+, CS−) were performed on affective valence and arousal ratings of the eight neutral faces. To evaluate whether potential changes in CS+/CS− valence and arousal were strong enough to bias ratings of emotional expressions (i.e., emotional transfer), separate repeated measures ANOVAs with the factors *Session* (Pre-conditioning, Post-conditioning), *CS Type* (CS+, CS−), and *Emotional Expression* (Angry, Neutral, Happy) were performed on valence and arousal ratings of all 24 face images.

Analyses of behavioral and evaluative data were conducted using SPSS Statistics (IBM). For all analyses in this article, we reported Greenhouse–Geisser corrected significance values, if the assumption of sphericity was violated.

## Results

### Skin conductance changes

**Figure 2:**
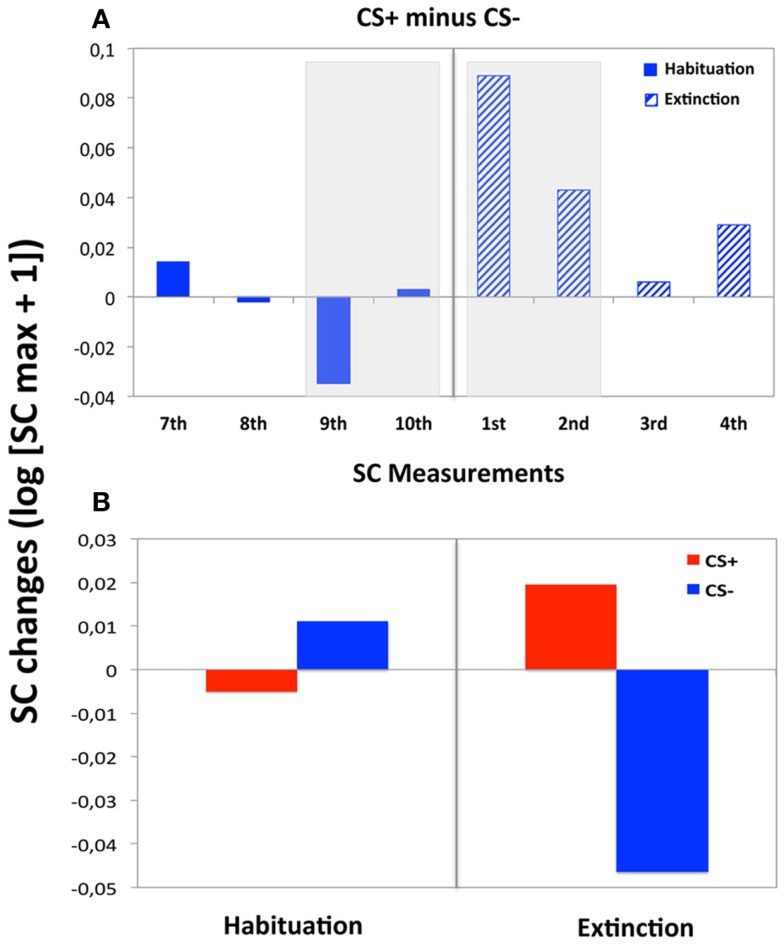
**Electrodermal reactivity during habituation and extinction**. **(A)** SC changes showing the electrodermal CS+/CS− differentiation across tested SC measurements in pre- and post-learning phases. In solid, dark blue, the last four SC measurements in habituation phase; in dashed, light blue the first four SC measurements during extinction trials. **(B)** Averaged electrodermal activity for the CS− and CS+, over the SC measurements selected for the repeated measures ANOVA described in this section (i.e., the last two SC measurements during habituation trials and the first two SC measurements during extinction).

The overall ANOVA showed that SC changes did not vary with CS Type, *F* < 1, but that the main effect of Phase was significant by trend, *F*(1, 33) = 3.86, *p* = 0.064, indicating greater SC responses during extinction (*M* = 0.19, SD = 0.22) compared to habituation (*M* = 0.14, SD = 0.19). Yet, the interaction Phase x CS Type did not reach significance, *F*(1, 33) = 1.75, *p* = 0.19. CS+ and CS− stimuli were compared separately for each SC measurement conducted during extinction to explore whether the expected electrodermal CS+/CS− differentiation was extinguished across trials after contingency removal, but still present at the beginning of this phase (see text footnote 1). To this extent, SC activity was greater for CS+ than for CS− during the first measurement of extinction, *t*(30) = −2.65, *p* < 0.05, and was still enhanced during the second measurement, *t*(30) = −2.17, *p* < 0.05, suggesting an effective associative learning after conditioning (Table 1). However, this CS+/CS− differentiation disappeared during the third SC measurement, *t*(30) = −0.36, *p* = 0.719, and remained absent during the fourth SC measurement, *t*(30) = −0.02, *p* = 0.209, or the additional measurements within this phase (Figure 2A). Thus, electrodermal CS+/CS− differentiation was measurable at trials 21/22 (i.e., up to around 3 presentations of each individual CS), but no longer at trials 39/40 (i.e., after 5 presentations of each CS). In addition, the repeated measures ANOVA across the first two SC measurements during extinction and the last two SC measurements during habituation yielded a significant effect for CS Type, *F*(1, 30) = 5.10, *p* < 0.05, with greater SC activity during CS+ (*M* = 0.01, SD = 0.08) than CS− (*M* = −0.02, SD = 0.08). The interaction of Phase × CS Type was also significant, *F*(1, 30) = 9.31, *p* < 0.005, suggesting differential SC activity after relative to before affective learning (Figure 2B). Indeed, *post hoc t-*tests performed separately in each phase showed that SC changes to CS+ (*M* = 0.02, SD = 0.10) were enhanced compared to CS− (*M* = −0.05, SD = 0.08) for the averaged first two extinction measurements, *t*(30) = −3.61, *p* < 0.005, but did not differ for the averaged last two habituation measurements, *t*(30) = 1.14, *p* = 0.262 (*M*s = 0.01, SDs = 0.06 and 0.05; for CS− and CS+, respectively).

**Table 1:** **Mean skin conductance changes (SE in parenthesis) during CS+ and CS−, for habituation and extinction SC measurements**.

	Habituation		Extinction	
	CS+	CS−	CS+	CS−
SC measurement first	0.020 (0.019)	0.006 (0.019)	0.025 (0.030)	−0.064 (0.026)
SC measurement second	0.021 (0.017)	0.023 (0.019)	0.014 (0.018)	−0.029 (0.006)
SC measurement third	−0.008 (0.015)	0.027 (0.020)	0.013 (0.012)	0.007 (0.011)
SC measurement fourth	−0.002 (0.005)	−0.005 (0.005)	0.004 (0.008)	−0.025 (0.024)

*SC measurements (first … fourth) correspond to the last four (measured) CS+/CS− during habituation, or the first four (measured) CS+/CS− during extinction, but do not refer to consecutive trials within the experimental design. The second ANOVA described in the main text was conducted with the average of two-measured CS+/CS− (third and fourth for habituation, first and second for extinction, according to this table)*.

### Electroencephalography

**Figure 3:**
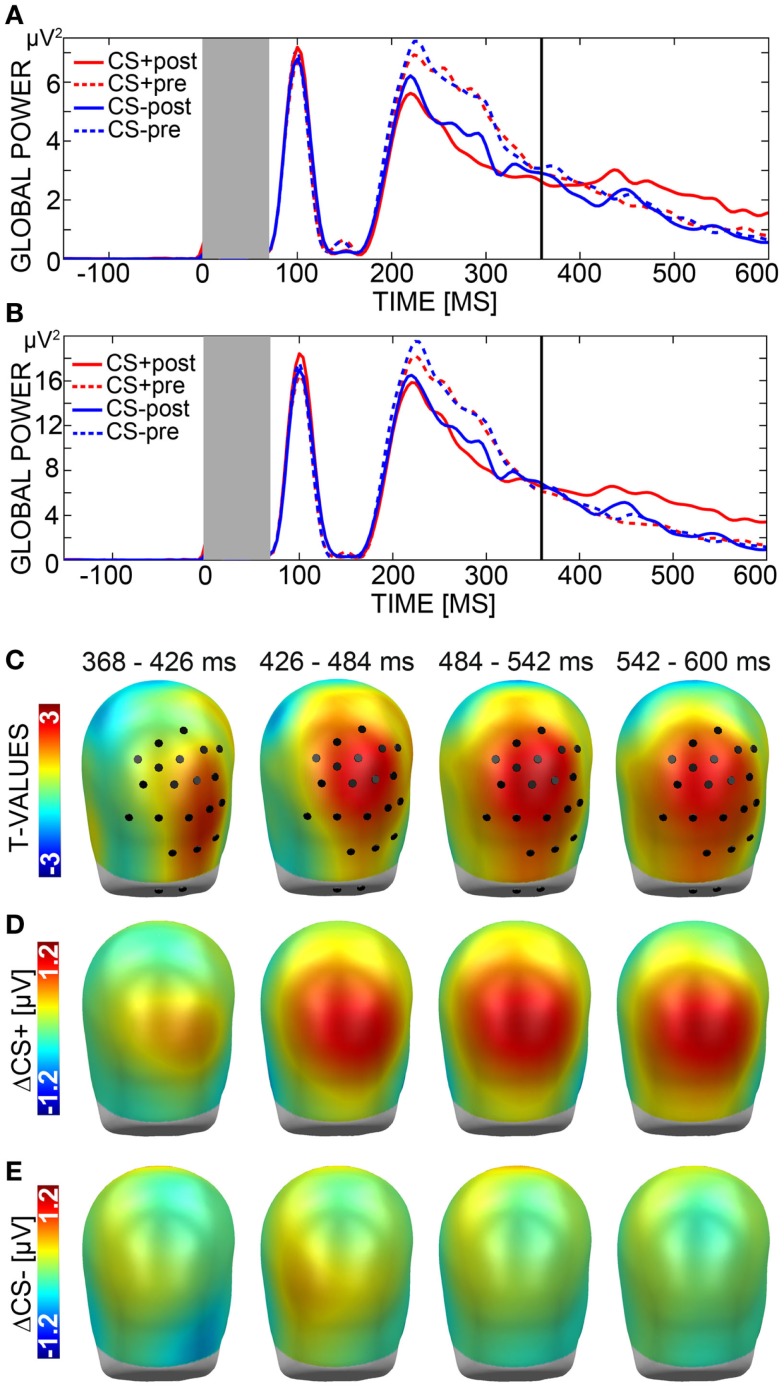
**Electroencephalography (EEG)**. **(A)** The global power plot shows the mean activation of all sensors in response to aversively paired (CS+) faces after (red solid line) and before (red dotted line) learning as well as in response to unpaired (CS−) faces after (blue solid line) and before (blue dotted line) learning from 150 ms before to 600 ms after stimulus onset. The dark gray box marks the time window (0–70 ms) in which a trigger-related technical artifact occurred. Analysis was carried out across a typical time window of the late positive potential (LPP) ranging from 300 to 600 ms. The starting point of the time window (368–600 ms) in which the significant effect emerged is marked by a bold line. **(B)** Global power plot displaying the mean activation across significant sensors only, with labeling being equivalent to A. **(C)** *t*-Values for the comparison of ΔCS+ (CS+_post_ minus CS+_pre_) and ΔCS− (CS−_post_ minus CS−_pre_) are plotted onto standard heads shown from back view. Black circles mark the sensor group displaying the significant effect during the selected time window (368–600 ms). **(D)** ΔCS+ and **(E)** ΔCS− are plotted onto standard heads shown from back view.

Analysis of the LPP yielded a significant effect with a cluster mass of 1203 (critical cluster mass: 763) over centro-posterior sensors in a time interval ranging from 368 to 600 ms after CS onset (Figures 3B,C). Visual analysis of topographies for ΔCS+ and ΔCS− confirmed the effect to signal relative positivity for ΔCS+ compared to ΔCS− (Figures 3D,E). Additional *post hoc t*-tests for this effect comparing CS+ and CS− activation measured during habituation and extinction indicated that CS+ faces evoked an enhanced positivity during extinction (*M* = 2.12, SD = 1.88) relative to habituation trials (*M* = 1.60, SD = 1.52), *t*(34) = 2.90, *p* = 0.006, while CS− amplitude remained constant (Habituation: *M* = 1.63, SD = 1.41; Extinction: *M* = 1.62, SD = 1.42), *t*(34) = 0.10, *p* = 0.922. Importantly, CS+ and CS− differed in amplitude over extinction, *t*(34) = 3.37, *p* = 0.002, but not over habituation trials, *t*(34) = −0.18, *p* = 0.859.

### Post-experimental contingency report

In the forced choice assignment performed after conditioning, 86.11% (SD = 15.17) of the CS+ were correctly paired with occurrence of a shock (UCS), whereas 92.36% (SD = 11.68) of all CS− was correctly paired with absence of a shock. Participants were by trend better at matching CS−/noUCS than CS+/UCS pairs, *t*(35) = 1.95, *p* = 0.059. Mean confidence did not differ between CS+ (*M* = 82.88, SD = 15.73) and CS− assignments (*M* = 78.68, SD = 22.16), *t*(35) = 1.55, *p* = 0.129. Sensitivity index *A*′ (*M* = 0.94, SD = 0.06) differed significantly from chance level (test value = 0.5), *t*(35) = 44.35, *p* < 0.001.

### Effects of contingency awareness on electrodermal reactivity and electroencephalography

In order to explore the above results in more detail, additional statistical tests were performed separately on skin conductance changes and EEG activity with two groups of participants (fully aware vs. unaware), based on their post-experimental reports concerning awareness of CS+/UCS relationship. Note that group sizes were highly imbalanced with substantially more aware (*N* = 31 for SCL and *N* = 35 for EEG analysis) than unaware (*N* = 9) participants.

For the electrodermal changes, we focused specifically on the repeated measures ANOVA across the first two SC measurements during extinction and the last two SC measurements during habituation, including the factor *Contingency Awareness* (see Figure 2B). Contingency awareness seemed to have an effect on skin conductance changes, as the Phase × CS Type × Contingency Awareness interaction reached trend-level significance, *F*(1, 37) = 3.36, *p* = 0.07. Similar results were found in the repeated measures ANOVA performed specifically on the averaged first two extinction measurements, which revealed a significant interaction for CS Type × Contingency Awareness, *F*(1, 37) = 4.74, *p* < 0.05. In addition, for participants unaware of the CS+/UCS relationship, no effects of CS Type or Phase × CS Type were found [*F*s(1, 37) < 1.69, *p*s > 0.24], suggesting an absence of electrodermal CS+/CS− differentiation with absent contingency awareness, both overall and after relative to before acquisition. To this extent, the *post hoc t-*tests performed separately in each phase showed that SC changes did not differ neither for the averaged last two habituation measurements, *t*(8) = −1.45, *p* = 0.187 (*M*s = −0.002 and −0.001, SDs = 0.006 and.004; for CS− and CS+, respectively), nor for the averaged first two extinction measurements, *t*(8) = 1.23, *p* = 0.255 (*M*s = −0.009 and −0.018, SDs = 0.032 and.018; for CS− and CS+, respectively), suggesting that sympathetic reactivity did not change across the experiment for the unaware participants.

Regarding cortical processing, however, additional statistical tests indicated that fully aware and not fully aware (i.e., unaware) participants showed a similar CS+ enhancement relative to CS− during the LPP time window. In detail, a two samples *t*-test was calculated, comparing the difference of ΔCS+ minus ΔCS− activation between the aware and unaware participants. Here, contingency awareness did not seem to influence the increase in CS+ activation after conditioning, as the *t*-test did not yield a significant difference between aware and unaware participants, *t*(42) = 0.08, *p* = 0.938.

### Subjective ratings of valence and arousal

#### CS+/CS− Differentiation

**Figure 4:**
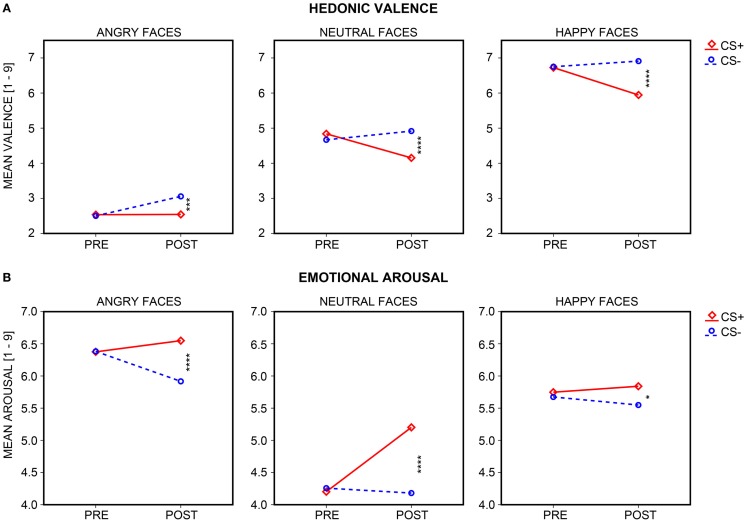
**Subjective ratings of hedonic valence and emotional arousal**. Mean **(A)** hedonic valence and **(B)** emotional arousal ratings acquired pre- and post-learning are shown for the neutral faces (central plot) which were paired (CS+; diamonds and solid red line) or remained unpaired (CS−; circles and dashed blue line) with UCS (electrical shocks) during the differential aversive conditioning procedure, as well as for the faces of CS+ and CS− identity with angry (left plot) or happy (right plot) expression. Stars depict the significance of the *t*-test comparing mean CS+ and CS− ratings after conditioning, with a greater number of stars depicting a significance value of *p* < 0.05, 0.01, 0.005, and.001, respectively.

Self-report data (hedonic valence, emotional arousal) are shown in Figure 4 (for descriptive statistics, see Table 2). Significant interactions of Session × CS Type emerged for both valence and arousal, *F*s(1, 35) = 29.39 and 16.27, *p*s < 0.001. CS+ decreased in valence and increased in arousal across sessions, *t*s(35) = −4.03 and 4.26, *p*s < 0.001, while CS− increased in valence and remained constant in arousal, *t*s(35) = 2.71 and 0.44, *p* < 0.05 and *p* = 0.662. There was no CS+/CS− differentiation prior to conditioning, *t*s(35) = 1.52 and −0.56, *p*s ≥ 0.137, but indeed after conditioning, as CS+ was rated as more unpleasant and more arousing than CS−, *t*s(35) = −5.13 and 3.99, *p*s < 0.001.

**Table 2:** **Overview of subjective valence and arousal ratings**.

	Angry faces		Neutral faces		Happy faces		Overall	
	CS+	CS−	CS+	CS−	CS+	CS−	CS+	CS−
**Valence**
Pre-conditioning	2.54 (0.76)	**2.50 (0.89)**	**4.84 (0.58)**	**4.67 (0.59)**	**6.72 (0.91)**	6.75 (0.88)	**4.70 (0.46)**	**4.64 (0.41)**
Post-conditioning	2.54 (0.97)	**3.06 (0.97)**	**4.15 (0.93)**	**4.92 (0.60)**	**5.94 (1.52)**	6.91 (0.90)	**4.21 (0.78)**	**4.96 (0.36)**
**Arousal**
Pre-conditioning	6.38 (1.13)	**6.38 (1.22)**	**4.20 (0.97)**	4.26 (1.05)	5.75 (1.09)	5.67 (1.11)	**5.44 (0.64)**	5.44 (0.64)
Post-conditioning	6.55 (1.28)	**5.92 (1.28)**	**5.20 (1.34)**	4.18 (1.37)	5.84 (0.97)	5.55 (1.03)	**5.86 (0.80)**	5.22 (0.87)

*Differences in pre- and post-conditioning valence and arousal ratings are assessed via paired *t*-tests and significant comparisons are marked in bold*.

*Means and SDs (in parenthesis) are displayed for hedonic valence and emotional arousal ratings, measured before and after conditioning in response to the four CS+ and four CS− faces (neutral expression) used during conditioning and the eight angry and eight happy faces of the same (CS+, CS−) identity*.

#### Emotional Transfer

Self-report data (hedonic valence, emotional arousal) are shown in Figure 4 (for descriptive statistics, see Table 2). Main effects of Emotional Expression for valence and arousal, *F*s(2, 70) = 230.23 and 41.45, *p*s < 0.001, confirmed that happy faces were rated as most pleasant, compared to neutral and angry faces, *t*s(35) = 12.06 and 16.73, *p*s < 0.001, and angry faces as least pleasant, in comparison to neutral and happy faces, *t*s(35) = −14.22 and −16.73, *p*s < 0.001. Both angry and happy faces were rated as more arousing than neutral faces, *t*s(35) = 7.91 and 7.12, *p*s < 0.001, and angry faces as more arousing than happy expressions, *t*(35) = 2.90, *p* < 0.010. Again, significant interactions of Session × CS Type emerged for valence and arousal, *F*s(1, 35) = 32.40 and 22.05, *p*s < 0.001. CS+ decreased in valence and increased in arousal across sessions, *t*s(35) = −3.95 and 5.22, *p*s < 0.001, whereas CS− increased in valence, *t*(35) = 5.25, *p* < 0.001, and decreased by trend in arousal, *t*(35) = −1.92, *p* = 0.063. There was no CS+/CS− differentiation in valence or arousal prior to conditioning, *t*s(35) = 0.77 and 0.05, *p*s ≥ 0.447, but after conditioning, with CS+ being rated overall as more unpleasant and arousing than CS−, *t*s(35) = −5.48 and 4.41, *p*s < 0.001. To this extent, we need to highlight that evaluative CS+/CS− differentiation after conditioning was not circumscribed to neutral faces (i.e., particularly to the ones used during the conditioning procedure), but it was also transferred to valence and arousal ratings of angry and happy faces of the same identities. More specifically, in all three facial expressions (angry, neutral, happy), CS+ were always rated as more unpleasant and arousing than CS− after the conditioning task, all *p*s ≤ 0.037, but not before aversive conditioning, all *p*s ≥ 0.137.

Furthermore, three-way Session × CS Type × Emotional Expression interactions reached trend-level significance for valence, *F*(2, 70) = 2.79, *p* = 0.071, and significance for arousal, *F*(2, 70) = 3.88, *p* = 0.033, which resulted from CS Type × Emotional Expression interactions on valence and arousal after, *F*s(2, 70) = 3.86 and 6.32, *p*s ≤ 0.032, but not before conditioning, *F*s(2, 70) = 1.04 and 0.24, *p*s ≥ 0.352. After aversive conditioning, the reduction in CS+ relative to CS− valence was equally pronounced in ratings of happy and neutral faces, *t*(35) = −1.38, *p* = 0.176, but greater for happy compared to angry faces, *t*(35) = −2.36, *p* = 0.024, and greater (by trend) for neutral compared to angry faces, *t*(35) = −1.70, *p* = 0.097. The increase in CS+ relative to CS− arousal after conditioning was greater in neutral compared to happy faces, *t*(35) = 3.20, *p* = 0.003 [neutral vs. angry: *t*(35) = 1.86, *p* = 0.071; angry vs. happy: *t*(35) = 1.94, *p* = 0.061]. Thus, regarding subjective hedonic valence, transfer of CS+/CS− emotionality occurred more easily to same identity faces with happy than with angry expressions, although floor effects for valence ratings of angry faces have to be taken into consideration. Regarding subjective arousal, this emotional transfer took place less easily to same identity faces with happy than with angry expression, although here ceiling effects might have been at play.

## Discussion

Rapid extinction of conditioned responding, overlearning of stimulus features, and difficulties in integration of both recording and analysis requirements for autonomous vs. central nervous system measures pose obstacles in classical conditioning research, which could be met by adjusting different study parameters. Here, we proposed to increase the number of conditioned stimuli – to limit the number of learning trials – and to use ITIs of different durations, whereas we evaluated a MultiCS conditioning set-up, in which the above adjustments were incorporated.

In accordance with previous classical conditioning studies [e.g., Pizzagalli et al. (2003)], we observed a centro-posterior positivity during a LPP time interval in the EEG, consistent with enhanced neuronal activation toward paired relative to unpaired stimuli after as compared to before learning. Enhanced positivity to CS+ was accompanied by greater electrodermal changes toward CS+ relative to CS−, but this increase was only observed during the first two measurements after conditioning. In addition, subjective affective ratings revealed that CS+ was evaluated as less pleasant and more arousing than CS− after relative to before conditioning. Moreover, conditioning-induced changes were strong enough to bias valence and arousal ratings of the CSs, even when they were shown with a different expression (i.e., happy and angry faces), but were indeed not used in the actual conditioning procedure. To this extent, it is important to note that most of the participants showed high awareness of CS+/UCS and CS−/noUCS contingencies in the post-experimental query. Notwithstanding, in order to be more confident of acquisition of “fear learning,” all the measures were analyzed here including only the fully aware participants.

The centro-posterior positivity for CS+ during the LPP appeared similar to the positivity found in a previous study, in which neuronal activation toward unreinforced presentations of CS+ and CS− was compared during partial reinforcement [cf., Pizzagalli et al. (2003)]. In such a partial reinforcement design, extinction of the CS+/UCS association is minimized because the non-appearance of a UCS after one CS+ presentation does not predict that the next CS+ will also remain unpaired. Thus, the similarity of the present EEG results and the ones reported by Pizzagalli et al. (2003) suggests that the incorporated adjustments hampered extinction, so that the continuous recall of the CS+/UCS association after learning elicited a sustained enhanced positivity for CS+. This interpretation is further corroborated by results of Steinberg et al. (2013a), which revealed that unreinforced CS presentations after the acquisition phase resulted in an extinction of mid-latency and late, but not early (<100 ms) CS+/CS− differentiation. The presence of a CS+/CS− differentiation during the LPP, as observed here, suggests that extinction was minimal or at least did not considerably reduce the degree to which the CS+ activated motivational circuits in the brain.

However, if the course of extinction was indeed decelerated at the cortical level, it seems surprising that the enhanced electrodermal changes to the CS+ disappeared after only a few trials of unreinforced CS+ presentations. This dissociation of sustained central, but diminished autonomous CS+/CS− differentiation could result from the strong dependence of conditioned SCRs on contingency awareness and UCS expectancy [see Lovibond and Shanks (2002) and Hamm and Weike (2005), for review]. For example, several studies have shown that enhanced autonomous responses to the CS+ are only found if participants are fully aware of CS+/UCS contingencies (Hamm and Vaitl, 1996; Weike et al., 2005, 2007; Pastor et al., 2013). Furthermore, some authors argued that enhanced electrodermal CRs to CS+ might signal both associative learning and formation of declarative memory rather than the acquisition of fear [e.g., Soeter and Kindt (2010)]. This argument is supported by studies that showed SCRs to be similarly enhanced in response to CS+ previously paired with an aversive UCS, as well as in response to CS+ previously paired with a non-aversive UCS (Lipp et al., 1994; Hamm and Vaitl, 1996; Weike et al., 2008). The fast disappearance of the enhanced electrodermal responses to the CS+ during extinction could thus indicate that participants learnt the new association of CS+/no UCS on a cognitive level and did not expect the UCS to occur after the CS+.

Interestingly, in a prior study focused on the effect of repetition on inherently emotional pictures, Codispoti et al. (2006) also found a rapid decay of enhanced SCRs, but a relative stability of an increased LPP to arousing relative to neutral pictures. They concluded that the LPP displayed automatic motivated attention toward salient, behaviorally relevant stimuli [cf., Lang et al. (1997)] and that the neuronal structures, which mediated this motivational processing were persistently recruited irrespective of stimulus repetition. They further suggested enhanced SCRs to indicate that the organism was orienting itself toward salient stimuli and preparing for action, but this orienting response disappeared rapidly across stimulus repetition, because the organism noticed that no action was required. Thus, the dissociation of neuronal and autonomous responding observed here could indicate that CS+ remained to be “salient stimuli” over the course of extinction, and thus received preferential neuronal processing. However, CS+ progressively ceased to pose an immediate threat to the organism after contingency removal, failing to elicit sustained response preparation, and consequently enhanced electrodermal changes [e.g., Vuilleumier (2005) and Bradley (2009)].

It seems important to note that the problem of extinction of emotional responses after conditioning could also be circumvented by assessing CS processing already during acquisition. For example, recent studies showed an enhanced sensory processing of CS+ vs. CS− during the learning of UCS/CS+ contingencies [e.g., Keil et al. (2013), Miskovic and Keil (2013), and Wieser et al. (2014)]. Although CS processing during acquisition can easily be assessed using EEG/MEG, measurement of skin conductance during learning is difficult to carry out simultaneously with the above measures due to the slow development (and recovery) of electrodermal responses. In the present study, the simultaneous presentation of the UCS (i.e., aversive shocks appeared 500 ms after CSs onset), together with the short duration of visual stimuli (i.e., CSs were presented only for 1000 ms), produced too many artifacts during CS+ trials, which made it difficult to analyze SC changes during the acquisition phase. In addition, it is necessary to highlight that according to our design, any plausible enhanced electrodermal activity for CS+ compared to CS− during acquisition trials would be basically reflecting a response to the aversive UCS instead of anticipatory responses cued by the CS+. The latter response, and not the pure reaction to the UCS, has been classically considered as a reliable index of “associative or relational learning” in differential aversive conditioning paradigms using peripheral correlates, where long CS presentation (i.e., 8 s) are generally followed by a long ITI (i.e., 12–15 s). Of course, a partial reinforcement design in which not all CS+ trials are reinforced during acquisition (and only unreinforced trials are analyzed) could have prevented the occurrence of artifacts. However, we propose that assessing activation during learning and assessing activation after learning provides different information about human fear processing, which might or might not be relevant to the investigated research question. More specifically, CS processing measured after relative to before learning reflects rather the recall than the encoding of emotional information. Furthermore, with UCSs being presented during acquisition but not extinction, different contexts are created in which the participant is set in a clearly threatening (acquisition) or potentially threatening (extinction) surrounding. Such changes in context may increase or decrease differential CS+/CS− activation within subjects, or even differences in CS processing between groups varying in individual difference variables.

Evaluative judgments revealed that participants rated CS+ faces as less pleasant and more arousing than CS−, when comparing after vs. before conditioning. Importantly, these changes in CS hedonic valence and arousal were remarkable as post-learning evaluative ratings were recorded after a long extinction session. Moreover, changes in hedonic valence and emotional arousal were not only confined to the originally conditioned neutral CS+ and CS− faces but also extended to different images showing faces of CS+ and CS− identity with angry and happy expressions. The most important implication of this “emotional transfer” is that participants learnt to associate not merely some low-level stimulus feature (such as head shape) with the occurrence or non-occurrence of an UCS, but acquired an association of facial identity and aversive events (presence of electrical shocks). Analyses revealed that valence ratings differed more between happy faces presented with positive CS− (i.e., safe) and negative CS+ (i.e., threat) association than between angry faces presented with the same positive or negative association. This result is surprising considering the concept of preparedness [e.g., Seligman (1970) and Öhman and Mineka (2001)] in which fear relevant as compared to non-relevant stimuli are more easily associable with aversive events. For example, angry relative to happy faces were shown to be more resistant to extinction, as measured with differential skin conductance and electromyographic responses, and evoked enhanced ratings of fear (Dimberg, 1987). Following this line of argumentation, the difference in valence between the angry CS+ and the angry CS− should have been greater than the difference between the happy CS+ and the happy CS−. That we observed the opposite pattern could be explained by taking into account investigations by Bradley and associates. For example, Bradley et al. (2005) showed fear-potentiated startle – regarded as an indicator of stimulus valence [e.g., Bradley et al. (1993)] – to vary in response to pleasant pictures presented in threatening and safe contexts, but to remain constant in response to unpleasant pictures presented in the same conditions. Their findings suggest that valence is more easily reducible for pleasant stimuli than for something already regarded as unpleasant, and that it is difficult (at least in an experimental setting) to make something unpleasant less unpleasant. However, it must be considered that floor effects might have prevented a decrease in CS+ valence for angry faces.

The major limitation of the present study concerns the occurrence of artifacts, which impeded analysis of earlier EEG components, such as the EPN. These artifacts probably resulted from the simultaneous use of different stimulation and recording systems, which relied on the introduction of external currents (skin conductance measure, electric shock) and several grounds (see text footnote 2). We thus recommend to investigators interested in the combination of skin conductance and EEG to use unique acquisition equipment for autonomous and central nervous system measures. To this extent, electrodermal reactivity could be complemented with fear-potentiated startle. In contrast to the LPP and SCRs, which are good physiological correlates of emotional arousal (sympathetic activation), fear-potentiated startle is regarded to be a reliable index of stimulus hedonic valence [e.g., Bradley et al. (1993)] – and appears to be less dependent on contingency awareness than conditioned autonomous reactions, even after a single learning trial (Hamm and Vaitl, 1996; Pastor et al., 2013). In addition, fear-potentiated startle – unlike skin conductance modulation – remains relatively stable across stimulus repetitions (Bradley et al., 1993), being more resistant to habituation, and thus especially meaningful when having longer extinctions (i.e., with a high number of trials to reliably measure EEG).

Taken together, our results show that classical conditioning with an increased number of CSs (i.e., eight) elicited sustained CS+/CS− differentiation over the course of extinction in neuronal and evaluative measures, but not in autonomous responses, even though the number of learning trials was strictly reduced. It was suggested that the implemented adjustments (i.e., increased number of CSs, reduced number of learning trials) minimized the degree to which the recruitment of motivational brain systems was extinguished as well as impeded the possibility of overlearning stimulus features. Simultaneous EEG and SC measurement presented with several difficulties, the most major of which being artifacts occurring due to the use of several different recording systems.

In a follow-up study, we plan to explore whether original stimulus valence and acquired or conditioned valence might interact (i.e., “emotional transfer”), and how inherent stimulus valence could be altered in a classical conditioning context (Rehbein et al., 2014a). MultiCS conditioning could be an interesting paradigm to focus on different time-points of acquisition or extinction (such as early vs. late acquisition or extinction trials), as it allows for multiple repetitions of CS+/CS− conditions (without repetition of individual stimuli), and thus for investigating CRs during even a single learning trial [cf., Rehbein et al. (2014b)]. Weak rather than strong learning situations have been proposed to be more useful in revealing individual differences of clinically and subclinically anxious participants in fear conditioning (Lissek et al., 2006; Beckers et al., 2013), and fear acquisition under strictly controlled awareness in MultiCS conditioning could provide such a weak situation. In this regard, future studies should clarify to which number of CSs the paradigm can be extended to assure sufficient contingency awareness as necessary condition for electrodermal CS+/CS− differentiation. To this extent, prior classical conditioning studies using longer intervals but less number of presentations have repeatedly reported that the CR (measured with SC changes) rapidly habituates after CS+/UCS contingency removal, because of which it is being considered a good measure of relational or associative learning [e.g., Hamm and Weike (2005)]. Notwithstanding, this reliable result regarding conditioned SC changes seems to depend, moreover, on the intrinsic CS aversive content, suggesting that biologically fear-relevant stimuli – paired with an aversive UCS – might be more resistant to extinction, even after non-occurrence of the UCS (Öhman et al., 1975; Öhman and Mineka, 2001; Poy et al., 2006a,b).

To this extent, past neuroscience research has often put emphasis either on central correlates or on peripheral correlates of learning, even when both measures were recorded simultaneously. However, it seems important to find ways of connecting the different methodologies, as in a combination or “equal emphasis” they can provide a much more detailed view on learning itself, besides a better understanding of individual differences and psychopathologies in learning. For example, past research has found abnormalities in classical conditioning, or neuronal activation patterns in general, in patients with psychological disorders, but such aberrations are hardly specific enough to be used as diagnostic tools [e.g., Gillihan and Parens (2011)]. More complex designs with combinations of different psychophysiological measures [e.g., Nelson et al. (2013)], as well as more individualized experimental investigations, could provide a solution to this problem in the near future.

## Conflict of Interest Statement

The authors declare that the research was conducted in the absence of any commercial or financial relationships that could be construed as a potential conflict of interest.

## Supplementary Material

The Supplementary Material for this article can be found online at http://journal.frontiersin.org/article/10.3389/fnhum.2015.00336/abstract

## Footnotes

^1^SC measurements were not consecutive, but distributed along the experimental sequence. Specifically, SC measurements during habituation corresponded to trial number 4, 21, 39, 55, 69, 83, 101, 119, 135, 149, for the CS−, and trial number 5, 22, 40, 56, 70, 84, 102, 120, 136, 150, for the CS+. In addition, SC measurements during extinction were as follows: trial number 3, 21, 39, 55, 69, 83, 101, 119, 135, 149, for the CS−, and trial number 4, 22, 40, 56, 70, 84, 102, 120, 136, 150, for the CS+. Therefore, time distance between CSs was similar during habituation and extinction, ranging between 14 and 18 trials, either for the CS+ or CS−conditions.

^2^The combination of EEG and SC measurement with visual and UCS stimulation unfortunately resulted in trigger-related stimulus artifacts occurring during the first 70 ms of CSs, when the trigger codes with 50 ms duration were sent by the software used to control visual stimuli presentation (see Figure S1 in Supplementary Material for an illustration of the artifact). The artifact was too small to be visually identifiable during recording or after averaging of individual EEG data, but it became visible in the Grand Mean across all subjects and was highly significant in the final statistical analysis. As seen in the Figure S1 in Supplementary Material, the artifact was mainly located at the EEG ground electrode at central parietal location, but expanded to surrounding electrodes. The ground electrode in the applied 129-sensor EGI system is positioned between the sensors Cz and Pz. As this ground electrode is not member of the 129 sensors (i.e., 128 sensors plus reference electrode), which detect the ERP topography, its position (marked by a yellow circle in the figure) occurred as a missing electrode right above electrode 62 (marked by a green circle) in the upper left head model. Artifact topographies of the affective conditions as well as their corresponding relevant differences are presented at 8 ms after stimulus onset (marked by the first of the two dashed lines) where the potential of electrode 62 indicates a distinct minimum. The topography shows a negativity surrounding electrode 62. It appears reasonable that the so-called polar average reference effect (PARE; Junghöfer et al., 1999, 2006) led to a slight polar topography displacement, so that the artifact originally maximal at the ground electrode appears as maximal at electrode 62. The artifact was maximally negative at 8 ms and maximally positive at 58 ms – both marked by dashed lines. The mean amplitude shift at 58 ms was comparable to the mean P100 amplitude at this electrode (~2.5 μV). Here, we assume that the trigger codes (50 ms duration), which were sent by the software used to control visual stimuli presentation, were mainly responsible for a shift of the common ground (all stimulation and detection apparatus were attached to the same ground), taking a small time delay of 8 ms between trigger and stimulus onset into consideration. It might be possible that differing binary coding for the four conditions (Pre learning CS+, Pre learning CS−, Post learning CS+, Post learning CS−) with different numbers of high and low bits affected the common ground differently, resulting in small, but condition-specific fluctuations in the EEG ground electrode activity. The different coding might have led to an interaction pattern with overall increasing amplitudes from pre to post learning but distinctly enhanced increase for the CS+ compared to CS−condition. However, while the Post minus Pre difference topographies still look like the main artifact topography, the double difference (right head model) reveals a distinct negativity at left occipital electrodes and a distinct overall left-negative right-positive asymmetry. The time course of this interaction (black dashed line) indicates that this interaction finds its maximum around 40 ms. This might lead to an alternative interpretation of the artifact: In several MultiCS conditioning MEG studies of neutral faces, our research group found indications for extremely early learning effects visible at occipital areas already around 30–40 ms after stimulus onset, which could be explained as earliest sensory learning effects of the magnocellular pathway [see, e.g., Discussion in Steinberg et al. (2013b)]. However, we are still gathering more supporting data for these extremely early effects, which will then be published elsewhere. Nevertheless, the occipital interaction visible here could in fact represent a real neural effect. As this effect is strong at ventral areas not completely covered by electrodes, the reference effect (PARE) might be responsible for a condition-dependent amplitude shift of the ground-electrode centered artifact topography (i.e., the trigger related change of the ground-artifact is mere consequence of the PARE, driven by early amplitude difference at ventral regions). Thus, it is unclear whether the early interaction effect represents a pure technical artifact or a mixture of artifact and a real interaction. Except for the lateral polarity shift, the early interaction effect shows clear similarities with the topography of the P1 and the early posterior negativity (EPN). Thus, whether the early interaction is treated as technical artifact or a mixture of technical and neural effects, a PCA-based consideration of this topography has different effects also on the P1 and EPN topography as well as on interaction effects in these time intervals. If treated as technical artifact, an EPN interaction in the expected direction occurs mainly at left occipital areas. If treated as mixture artifact, we find this EPN interaction mainly at right occipital areas. This uncertainty about the rational of the artifact in connection with the not yet published 30–40 ms effects founded our decision to restrict the EEG analysis to the LPP interval because this sustained component reflected stable learning-related changes in CS+/CS− processing beyond doubt. As the condition-specific interaction in meaningful direction might confuse some readers who regard the figures only or do not read this rather complicated technical discussion, we decided to mask the affected time-interval between 0 and 70 ms in Figures 3A,B.
