# Supplementary material for: Facing Challenges in Differential Classical Conditioning Research: Benefits of a Hybrid Design for Simultaneous Electrodermal and Electroencephalographic Recording
Source: Front Hum Neurosci. 2015 Jun 9;9:336. doi: 10.3389/fnhum.2015.00336 (PMC4460875; doi:10.3389/fnhum.2015.00336)
Supplement: Supplementary file 1 [file image_1.pdf]

Supplementary Material

Facing challenges in differential classical conditioning research: Benefits of a hybrid design for simultaneous electrodermal and electroencephalographic recording

Maria Carmen Pastor[1]†, Maimu A. Rehbein[2,3]†, Markus Junghöfer[2,3]*, Rosario Poy[1], Raul López[1], Javier Moltó[1]

[1]Department of Basic and Clinical Psychology, and Psychobiology, Universitat Jaume I, 12071 Castellón, Spain
[2]Institute for Biomagnetism and Biosignalanalysis, University Hospital Münster, 48149 Münster, Germany
[3]Otto Creutzfeldt Center for Cognitive and Behavioral Neuroscience, University of Münster, 48151 Münster, Germany

*** Correspondence:** Markus Junghöfer, Institute for Biomagnetism and Biosignalanalysis, University Hospital Münster, Malmedyweg 15, 48149 Münster, Germany. Email: markus.junghoefer@uni-muenster.de.

1. Supplementary Figure

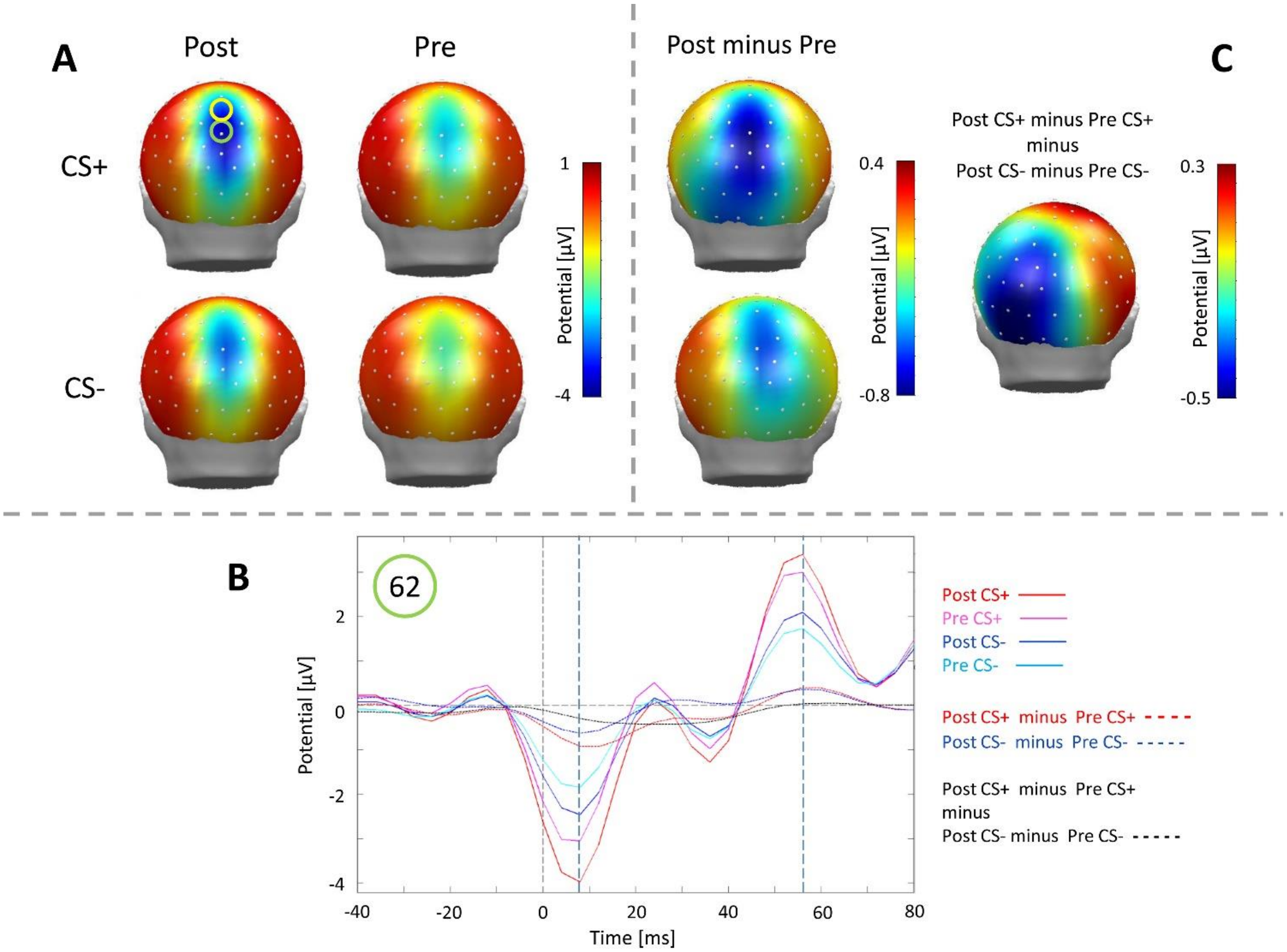

Supplementary Figure 1. Illustration of the artifact observed in electroencephalographic recordings. (A) Artifact topographies are displayed for CS+ (top) and CS- (bottom) during habituation (pre; right column) and extinction (post; left column) phases, projected onto standard heads shown from back view and with electrodes marked as white cylinders. The artifact was mainly located at the EEG ground electrode (yellow circle). In the topography, however, the electrode number 62 (green circle) located just below the ground electrode shows the maximal artifact probably due to the polar average reference effect (PARE; Junghofer et al., 1999; 2006). (B) Mean activation of CS+ (solid red and pink lines, respectively) and CS- (solid dark and light blue lines, respectively) during extinction (post) and habituation (pre) are displayed for electrode number 62, as well as the CS+ (red dashed line) and CS- (blue dashed line) difference activations (post minus pre) and the double difference thereof (black dashed line). The artifact was maximally negative at 8 ms and maximally positive at 58 ms – both latencies marked by dashed lines. The mean amplitude shift at 58 ms was comparable to the mean P100 amplitude at this electrode (~2.5 µV). (C) Artifact topographies are displayed for the CS+ (top left column) and CS- (bottom left column) difference activations as well as the double difference (right column).
